# 3D-Printable PP/SEBS Thermoplastic Elastomeric Blends: Preparation and Properties

**DOI:** 10.3390/polym11020347

**Published:** 2019-02-17

**Authors:** Shib Shankar Banerjee, Stephen Burbine, Nischay Kodihalli Shivaprakash, Joey Mead

**Affiliations:** Nanomanufacturing Center, Department of Plastic Engineering, University of Massachusetts Lowell, 1 University Avenue, Lowell, MA 01854, USA; Stephen_Burbine@student.uml.edu (S.B.); nischay_kodihallishivaprakash@student.uml.edu (N.K.S.)

**Keywords:** 3D printing, material extrusion, fused deposition modeling, thermoplastic elastomeric materials, morphology, properties

## Abstract

Currently, material extrusion 3D printing (ME3DP) based on fused deposition modeling (FDM) is considered a highly adaptable and efficient additive manufacturing technique to develop components with complex geometries using computer-aided design. While the 3D printing process for a number of thermoplastic materials using FDM technology has been well demonstrated, there still exists a significant challenge to develop new polymeric materials compatible with ME3DP. The present work reports the development of ME3DP compatible thermoplastic elastomeric (TPE) materials from polypropylene (PP) and styrene-(ethylene-butylene)-styrene (SEBS) block copolymers using a straightforward blending approach, which enables the creation of tailorable materials. Properties of the 3D printed TPEs were compared with traditional injection molded samples. The tensile strength and Young’s modulus of the 3D printed sample were lower than the injection molded samples. However, no significant differences could be found in the melt rheological properties at higher frequency ranges or in the dynamic mechanical behavior. The phase morphologies of the 3D printed and injection molded TPEs were correlated with their respective properties. Reinforcing carbon black was used to increase the mechanical performance of the 3D printed TPE, and the balancing of thermoplastic elastomeric and mechanical properties were achieved at a lower carbon black loading. The preferential location of carbon black in the blend phases was theoretically predicted from wetting parameters. This study was made in order to get an insight to the relationship between morphology and properties of the ME3DP compatible PP/SEBS blends.

## 1. Introduction

Currently, additive manufacturing (AM) or rapid prototyping (RP), commonly referred to as 3D printing, has gained enormous attention in the scientific community as a versatile manufacturing technique due to its inherent ability to print unique and complex geometries using computer aided design [1,2,3,4,5]. Among various other 3D printing technologies, such as selective laser sintering (SLS), direct ink writing (DIW), stereolithography (SLA), digital light processing (DLP), and polyjet, material extrusion 3D printing (ME3DP) based on fused deposition modeling (FDM) technique is considered by far the most versatile technology due to its low cost and convenient production of parts involving complex geometries [6,7,8,9]. In FDM technology, a thermoplastic monofilament is heated to its melting point inside a nozzle and deposited in a layer-by-layer approach at predefined positions onto a build-platform. The flexibility of FDM makes it an attractive manufacturing technique. However, the major limitation of FDM is the dependence on amorphous polymeric materials as a feedstock and, accordingly, it suffers from a limited number of thermoplastic choices. While the FDM process for a few thermoplastic materials has been well demonstrated, there still exists a significant challenge to develop compatible and new polymeric materials for ME3DP. To date, there are limited studies on this subject. The blending of two polymers is a straightforward approach to increase the number of ME3DP-compatible polymeric materials to widen the applications for 3D printing.

Thermoplastic elastomers (TPEs) are hybrid materials, generally made of thermoplastics and elastomers [10,11,12]. They are intriguing and fascinating specialty polymers, that have attracted the interest of the scientific community due to their unique elasticity and thermoplastic-like processability [13]. They have been used in a myriad of applications including automotive profiles, window gaskets, tubes, seals, electrical wires, etc. [14,15]. However, the development of products from TPEs is dependent on the cost of the mold, that becomes more expensive based on the design and complexity of the parts. In order to enrich the design and fabrication flexibility, there is a significant need to develop 3D printable TPEs.

The objective of this work was to develop 3D printable thermoplastic elastomeric materials from polypropylene (PP) and styrene-(ethylene-butylene)-styrene (SEBS) based on a blending approach. This blend was implemented to take advantage of the printability of PP and the elasticity of SEBS. PP is widely used for various commodity and industrial applications due to its low cost, excellent processability, mechanical properties, recyclability, etc. [16]. On the other hand, SEBS is an important polymeric elastomer, with high elongation at break, low processing temperature, low melt viscosity, and low distortion during extrusion [17,18]. Therefore, the blending of PP with SEBS is expected to provide new PP/SEBS thermoplastic elastomeric materials for 3D printing with a wider range of properties, such as enhanced elastomeric behavior and processability. Controlling the thermoplastic elastomer content in the blends enables the user to tailor the flexibility and elasticity of the resulting material, as well as the printed part, expanding the range of potential applications.

The influence of blend ratio on printability and properties was investigated. In order to improve the mechanical and dynamic mechanical properties of 3D-printed TPEs, reinforcing carbon black was used. The processability of 3D-printable TPEs was predicted from rheological measurements. Field emission scanning electron microscopy was used to investigate the morphology of the samples. In addition, the formation of droplet-matrix morphology of this blend was validated from Kerner’s two phase theoretical model [16]. In case of the filled TPEs, preferential location of carbon black was also theoretically predicted from wetting parameters. Finally, the properties of the 3D printed TPE were compared with traditional injected molded samples. This investigation was made to gain insight into the differences in the morphology and properties of the 3D printed PP/SEBS blends as compared to injection molded samples.

## 2. Materials and Methods

**Figure 1:**
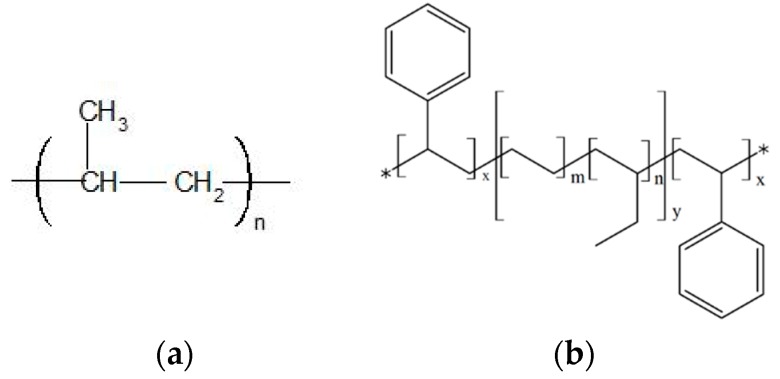
Chemical structure of (**a**) polypropylene (PP) and (**b**) styrene-(ethylene-butylene)-styrene (SEBS).

Isotactic polypropylene (PP 1345Z) having a density of 0.90 g/cm^3^, melting temperature of 165 °C and a melt flow rate of 45 g/10 min (230 °C and 2.16 kg) was kindly supplied by Pinnacle Polymers (Garyville, LA, USA). SEBS (G 968 SEBS) having a density of 0.89 g/cm^3^ was obtained from Mexichem (Tlalnepantla, State of Mexico, Mexico). Reinforcing carbon black, N330 with a STSA surface area of 76 m^2^/g was procured from Cabot (Boston, MA, USA). The chemical structure of PP and SEBS is given in Figure 1.

The PP/SEBS blends in the composition of 20 to 80 wt % of SEBS were prepared by melt blending in a twin screw extrusion process (Leistritz ZSE18HP-40D, l/d = 40). The parameters of the extrusion process are given in Table 1. One blend composition (40 PP/60 SEBS, *w*/*w*) which showed thermoplastic elastomeric behaviour was mixed with varying loading of carbon black in the range of 0 to 15 parts per hundred rubber (phr). In order to stabilize the blends during extrusion, a small amount of thermal stabilizer (Irganox HP 2215FF) was used in the amount of 0.5 wt % based on the weight of PP in the blend. The diameter of extruded monofilament for the 3D printing process was maintained at around 2.8 ± 0.1 mm.

### 2.1. 3D Printing

**Figure 2:**
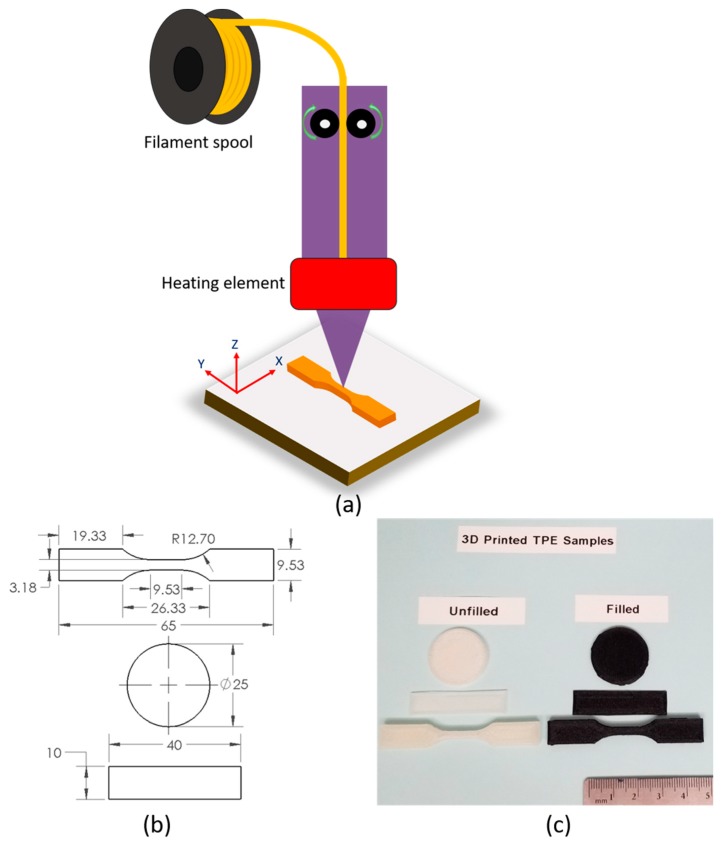
(**a**) Typical scheme of the 3D printing process, (**b**) drawing of specific sizes and shapes of the samples (tensile test, rheological test, and dynamic mechanical test) for 3D printing. All units are in mm. (**c**) 3D printed thermoplastic elastomers (TPEs) samples from 40 PP/60 SEBS (*w*/*w*) blend composition (unfilled and carbon black filled).

All test filaments were printed using the Ultimaker 3 (Cambridge, MA, USA) material extrusion 3D printer in order to verify commercial printability. The typical printing process can be seen in Figure 2a. The diameter of extruded monofilament was 2.8 ± 0.1 mm. Adhesion sheets were used in the build plate to prevent warping of the PP. The tensile specimens were printed as per the ASTM D638 standard. The samples for rheological characterization were printed as circular discs in accordance with the DIN EN 60404-5 standard with a diameter of 25 mm and a height of 2 mm. The rectangular specimens were printed with the dimension 40 (length) × 10 (width) × 1 (thickness) mm^3^ for dynamic mechanical analysis. The drawing of these specific sizes and shapes is shown in Figure 2b. The 3D printing process parameters of PP/SEBS blends are listed in Table 2. In order to maintain uniformity of each layer, all printing was done in a laid-down orientation. Figure 2c shows the unfilled and carbon black filled 3D printed TPE samples. 3D printed specimens were prepared in the X–Y axis to obtain the best mechanical properties from FDM method. It is important to mention here that the 20 PP/80 SEBS composition was not printable due to filament buckling inside the feeder as this composition was too soft compared to other blend compositions.

### 2.2. Mini-Injection Molding

Test specimens were also prepared using a mini-injection molding machine (Daca Instruments Model: 50000, Santa Barbara, CA, USA) for one blend composition (40 PP/60 SEBS, *w*/*w*) which showed the thermoplastic elastomeric behavior in order to compare the morphology and properties of injection molded TPE samples with respect to the 3D printed ones. The cylinder temperature and mold temperature were 220 and 50 °C, respectively. Injection pressure and holding pressure were 50 and 30 MPa, respectively. The injection and holding times were 15 s each.

### 2.3. Characterization

#### 2.3.1. Mechanical Testing

Mechanical properties were conducted according to ASTM D638 on dumb-bell shaped specimens using an Instron universal tester (Model: 4481, Instron, Norwood, MA, USA) at a constant crosshead speed of 100 mm/min. Dumb-bell shaped specimens were prepared using a 3D printer and an injection molding process. Young’s modulus was calculated as between 0.05 % and 0.25 % of strain.

Tension set was measured using an Instron tensile testing machine at room temperature. At first, samples were extended up to 100% elongation at 100 mm/min in the tensile direction and kept at that position for 10 min. Following this, the sample was removed and kept for 24 h for complete relaxation to return to unstressed conditions. The tension set value (change in dimension) was measured in the tensile direction after 24 h.

#### 2.3.2. Dynamic Mechanical Analysis

The dynamic mechanical properties of the samples (3D printed and injection molded) were analyzed using a DMA (model Q800, TA instrument, New Castle, DE, USA) in tension mode. All the samples were tested at a constant frequency of 1 Hz, a heating rate of 2 °C/min, and a strain value of 0.1% in the temperature range of −100 °C to +100 °C. Storage modulus (E′), loss modulus (E′′), and loss tangent (tanδ) were measured and analyzed as a function of temperature.

#### 2.3.3. Rheological Testing

The rheological measurements were performed using a parallel plate Rheometer (ARES G-2-TA Instruments, New Castle, DE, USA) having a diameter of 25 mm and a gap of 2.0 mm between the plates. Frequency sweep experiments were carried out at 220 °C in the frequency range of 0.1 to 100 rad/s at a constant strain of 5%. Storage modulus (G′), loss modulus (G′′), and complex viscosity (η*) were measured and analyzed as a function of frequency.

#### 2.3.4. Filed Emission Scanning Electron Microscopy

Field Emission Scanning Electron Microscopy (FESEM) (Model: JSM-7401F, JEOL, Akishima, Tokyo, Japan) was used to record the phase morphology of 3D printed and injection molded TPEs. The acceleration voltage was 3.0 kV at a working distance of 6 mm. A thin layer of gold was sputter coated for 180 s on the smooth sample surface to avoid charging on exposure to electron beam during the FESEM experiment.

In order to understand the dimension of the dispersed SEBS phase in the injection molded and 3D printed TPEs, the samples from each process were preferentially etched with the solvent, THF for 24 h at room temperature. The etched samples morphology was recorded using the FESEM.

## 3. Results and Discussion

### 3.1. Mechanical Properties

**Figure 3:**
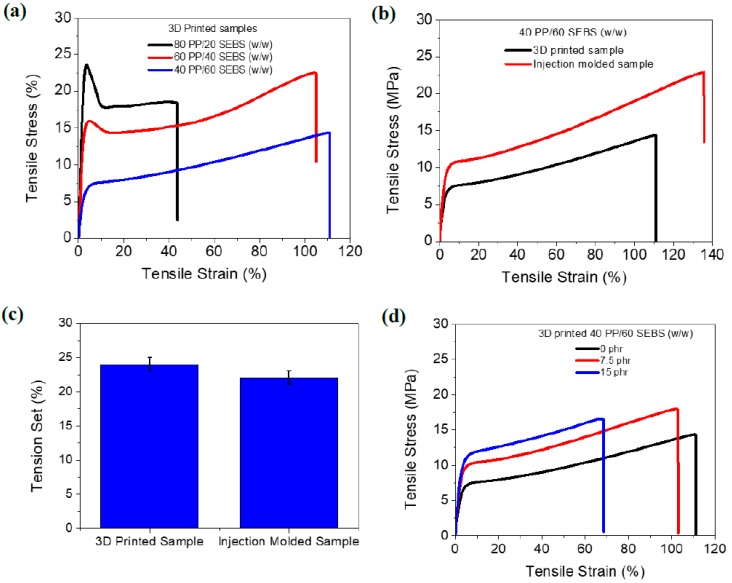
Mechanical properties of 3D printed blends: (**a**) Tensile stress vs. strain of 3D printed PP/SEBS blends having various blend ratios, (**b**) tensile stress vs. strain of 3D printed vs. injection molded TPE, (**c**) tension set of 3D printed vs. injection molded TPE, (**d**) tensile stress vs. strain of carbon black filled 3D printable TPE (loading of carbon black was varied from 0 to 15 parts per hundred rubber (phr)).

Mechanical properties of the 3D printed samples having compositions of 20 to 60 wt % SEBS are presented in Figure 3a–d. It was observed that the tensile strength and Young’s modulus increased with an increase in PP content. On the other hand, elongation at break increased with an increase in SEBS content. Differences in the stress–strain behavior among the different 3D printed compositions were clearly visible in their plastic deformation zones. Both the 80 PP/20 SEBS (*w*/*w*) and 60 PP/40 SEBS (*w*/*w*) blend compositions exhibited a narrow yield point along with a high modulus (Figure 3a). In addition, a consistent plastic deformation plateau was observed in both cases consistent with the behavior of thermoplastics. Interestingly, the curve of 40 PP/60 SEBS (*w*/*w*) composition had no yield point before failure, which is indicative of typical thermoplastic elastomer behavior (Figure 3a). Therefore, it is inferred that only the 40 PP/60 SEBS (*w*/*w*) blend composition can be considered a TPE when compared with the other two compositions. This finding is in line with the reported TPE literature [11]. Recently, Banerjee et al. prepared nanostructured TPEs from polyamide 6 and fluoroelastomer and they found 40 polyamide 6/60 fluoroelastomer (*w*/*w*) composition had the best thermoplastic elastomeric behavior compared with the other various blend compositions [11]. This 40 PP/60 SEBS (*w*/*w*) blend composition was used to compare 3D printed to injection molded samples.

It is well known that 3D printed parts are generated from layer-by-layer assembly technology. Parts produced in this manner are, therefore, expected to have lower mechanical performance as compared to injection molded samples. Figure 3b provides the mechanical performance of the 3D printed vs. injection molded TPEs. As expected, tensile strength, Young’s modulus, and elongation at break of the 3D printed TPE was lower than that of the injection molded samples. For example, tensile strength and Young’s modulus of the 3D printed TPE was ~14 and ~90 MPa, respectively. These values can be compared to the values of ~23 and ~150 MPa in the case of the injection molded TPEs. However, the tension set values were similar between the 3D printed part and the injection molded TPE (Figure 3c). Lower mechanical performance of 3D printed TPEs was the result of delamination of the printed layers due to the weak interface between the layers, which is quite challenging to improve.

To address this problem and create a stronger 3D printable material with higher mechanical performance, this work investigated the use of reinforcing carbon black in the TPE matrix. As seen in Figure 3d, at lower loadings of carbon black (7.5 phr) the tensile strength and Young’s modulus were increased with a slight reduction of the elongation at break value. Tensile strength, Young’s modulus, and elongation at break of the 7.5 phr loaded TPE were ~18 MPa, ~120 MPa, and 105%, respectively. The values could be compared to ~14 MPa, 90 MPa, and 110%, respectively for the TPE without carbon black. In contrast, at 15 phr loading of carbon black, the Young’s modulus was enhanced; however, the tensile strength and elongation at break were lower as compared to the 7.5 phr loaded TPE. Additionally, the 15 phr loading composition should not be classified as a TPE because the elongation at break was lower than 100%. Therefore, the lower loading of reinforcing carbon black was beneficial to improve the overall thermoplastic elastomeric properties and mechanical properties of a 3D printable TPE.

### 3.2. Dynamic Mechanical Properties

**Figure 4:**
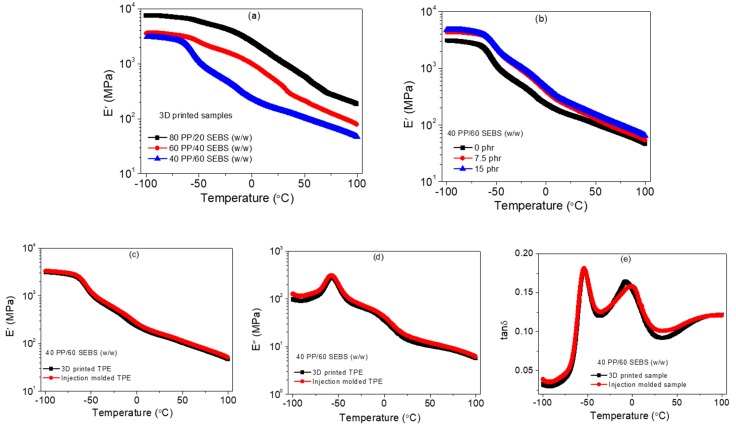
Dynamic mechanical properties of the blends in the temperature range of −100 to +100 °C: (**a**) Storage modulus (E′) vs. temperature of 3D printed PP/SEBS blends, (**b**) storage modulus (E′) vs. temperature of carbon filled loaded 3D printed TPE (loading of carbon black was varied from 0 to 15 phr) (**c**) storage modulus (E′) vs. temperature of 3D printed TPE vs. injection molded TPE, (**d**) loss modulus (E′′) vs. temperature of 3D printed TPE vs. injection molded TPE, and (**e**) loss tangent (tanδ) vs. temperature of 3D printed TPE vs. injection molded TPE.

Dynamic mechanical performance of the 3D printed samples was evaluated and compared with the injection-molded samples. The storage modulus (E′) of the 3D printed blends as a function of temperature is given in Figure 4a. By increasing PP content, the storage modulus of the blend increased, consistent with tensile properties. Figure 4b shows the dynamic mechanical behavior of the carbon black loaded TPEs. The E′ of the 3D printed TPE at 30 °C was ~140 MPa, which increased to ~185 MPa after the incorporation of 7.5 phr carbon black. At a 15 phr loading of carbon black, a slight increase in the storage modulus was obtained as compared with the 7.5 phr loading. The dynamic mechanical properties of the 3D printed TPE was compared with the injection molded TPE. There were no significant differences in the dynamic mechanical behavior in the temperature range of −100 to +100 °C. E′ and E′′ of the 3D printed TPE at 30 °C were 140 ± 6 and 12 ± 3 MPa, respectively. These values were 148 ± 6 and 15 ± 3 MPa in the case of the injection molded TPE. Interestingly, a slight reduction of the loss tangent peak and a small shift in the glass transition temperature of the PP phase at higher temperatures were observed for the injection molded TPE, possibly due to differences in thermal history. However, there was no change in the glass transition temperature of the SEBS phase for both processes.

### 3.3. Rheology

**Figure 5:**
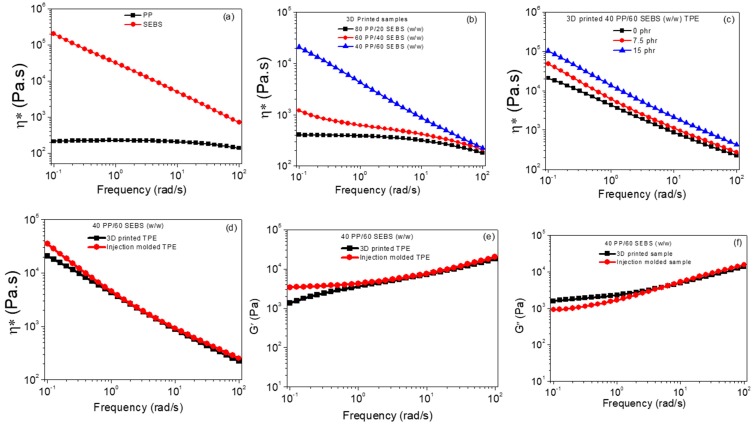
Rheological behavior of 3D printed PP/SEBS blends: (**a**) Complex viscosity (η*) vs. angular frequency of pristine PP and SEBS, (**b**) complex viscosity (η*) vs. angular frequency of 3D printed PP/SEBS blends having various blend ratios, (**c**) complex viscosity (η*) vs. angular frequency of the 3D printed carbon black filled TPE (loading of carbon black was varied from 0 to 15 phr) (**d**) complex viscosity (η*) vs. angular frequency of the 3D printed TPE vs. injection molded TPE, (**e**) storage modulus (G′) as a function of angular frequency of 3D printed TPE vs. injection molded TPE, and (**f**) loss modulus (G′′) as a function of angular frequency of 3D printed vs. injection molded TPE.

A rheological investigation of TPEs was necessary to optimize the processing conditions as the properties could depend on processing operations [19,20]. The viscosity behavior of the pure PP and SEBS, and their blends were examined using melt rheology as shown in Figure 5. Each experiment was performed at least three times from the same composition. The representative flow curve is provided here. The experimental standard deviation of complex viscosity and dynamic moduli were in the range of 3% to 4%. SEBS had a higher value of complex viscosity than PP in the entire frequency range tested (0.1 to 100 rad/s). Therefore, the lower viscosity PP would have a tendency to form the matrix in the PP/SEBS blends (discussed in the forthcoming section). Interestingly, SEBS had more shear thinning behavior than PP. This was due to more orientation and disentanglement of SEBS chains than PP with increasing shear rate [18]. All samples showed non-Newtonian shear thinning behavior. The frequency sweep curves of the printable blend samples are shown in Figure 5b–f.

The complex viscosity of the blend enhanced with an increase in SEBS content. Viscosity was further enhanced after incorporation of carbon black into the blend and also increased with carbon black loading. Finally, distinct melt rheological behavior of the TPE was investigated with special reference to the 3D printing and injection molding processes. Melt-rheological properties of the 3D printed TPE were critically compared with the injection molded TPE in terms of complex viscosity, storage modulus, and loss modulus. Interestingly, at a higher frequency range (1.00 to 100 rad/s), there was no significant change in the complex viscosity and dynamic moduli of the 3D printed TPE as compared with the injection molded TPE. However, at a low frequency range (0.1 to 1.0 rad/s), complex viscosity and storage modulus were slightly enhanced for the injection molded TPE compared to the 3D printed TPE. On the other hand, a slight reduction in the loss modulus was observed for the injection molded TPE at low frequency. The enhanced complex viscosity and dynamic moduli of the injection molded TPE might be the result of differences in morphology, such as a slight reduction in the domain size of the dispersed phase in the case of injection molded TPE as compared to the 3D printed TPE (discussed in the forthcoming section). In addition to these morphology differences, voids in the printed parts could also be responsible for the lower complex viscosity and dynamic moduli compared to the injection molded part.

### 3.4. Morphology

**Figure 6:**
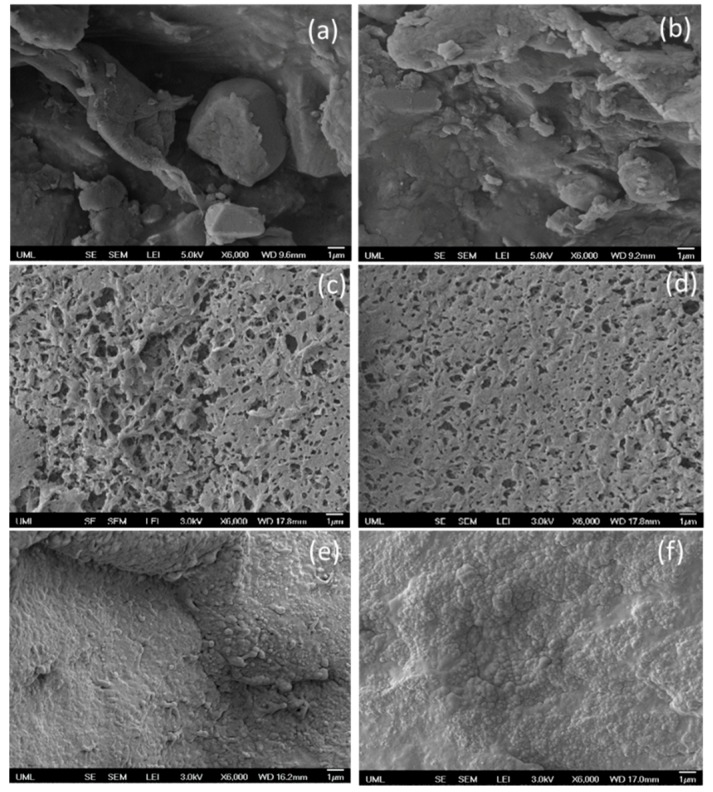
Morphology as observed by field emission scanning electron microscopy (FESEM) for (**a**) cryo-fractured surface of 3D printed 40 PP/60 SEBS (*w*/*w*) binary blend, (**b**) cryo-fractured surface of injection molded 40 PP/60 SEBS (*w*/*w*) binary blend, (**c**) etched morphology of the 3D printed 40 PP/60 SEBS (*w*/*w*) binary blend, (**d**) etched morphology of the injection molded 40 PP/60 SEBS (*w*/*w*) binary blend, (**e**) cryo-fractured surface of 7.5 phr carbon black loaded 3D printed 40 PP/60 SEBS (*w*/*w*) binary blend, and (**f**) cryo-fractured surface of 15 phr carbon black loaded 3D printed 40 PP/60 SEBS (*w*/*w*) binary blend.

It is well known that the morphology of polymer blends depend on various parameters, such as the viscosity ratio of blend components, viscoelastic properties of the components, processing conditions, shear rate, interfacial tension, capillary number, etc. [21,22] The morphology of PP/SEBS blends shown in Figure 6a,b clearly depict two distinct phases irrespective of the sample fabrication techniques, 3D printing and injection molding. As the polymer blend components, PP and SEBS were thermodynamically immiscible due to their high molecular weight and interfacial tension. Upon mixing, PP/SEBS blends undergo phase separation and, due to the viscosity mismatch, the low viscosity PP forms the continuous matrix phase and the SEBS is dispersed within the matrix. The droplet-matrix morphology was further validated using a theoretical model (discussed in the forthcoming section). In order to investigate the droplet size, SEBS was selectively etched using THF solvent and the resulting morphology is presented in Figure 6c,d, showing the two phase morphology very clearly. The pore size was quite large for the 3D printed sample as compared to that of the injection molded sample. This could be due to the high shear applied during the injection molding process, which reduces the droplet size of the dispersed phase. Interestingly, this morphology had a direct influence on mechanical properties of the resulting samples. Figure 3b, clearly shows the superior tensile strength, Young’s modulus, and elongation at break for the injection molded sample (smaller droplets) when compared to those of the 3D printed one.

Introducing reinforcing fillers such as carbon black is a widely practiced strategy to compensate for the loss of mechanical properties. In order to improve the mechanical performance of the 3D printed sample, reinforcing carbon black at 7.5 and 15 phr loading was incorporated into the TPE matrix. The resulting morphology is shown in Figure 6e,f. Interestingly, on addition of carbon black particles, the two phase morphology became quite challenging to observe. As seen from Figure 6e,f, the carbon black particles were fairly well dispersed in the TPE matrix. However, a few agglomerated structures of carbon black were seen in the case of the 15 phr loaded sample. The location of carbon black particles in the TPE blend phases was predicted by the wetting parameter (ω_α_) as given below [23,24,25]:

(1) $$\omega_{\alpha }=\frac{\gamma_{\mathrm{CB}/\mathrm{SEBS}}-\gamma_{\mathrm{CB}/\mathrm{PP}}}{\gamma_{\mathrm{PP}/\mathrm{SEBS}}}$$

where γ_CB/SEBS_, γ_CB/PP_, γ_PP/SEBS_ were the interfacial tensions between carbon black and SEBS, carbon black and PP, PP and SEBS phases, respectively. Interfacial tension was obtained theoretically from surface tension values of the dispersion and polar components. [26] When ω_α_ < −1, carbon black particles should preferentially move to the SEBS phase, and when ω_α_ > 1, the opposite trend should be observed where carbon black particles prefer to stay in the PP phase.

The interfacial tension between the phase components was calculated according to Wu as given below [26,27]:

(2) $$\gamma_{1-2}=\gamma_{1}+\gamma_{2}-2\sqrt{\gamma_{1}^{d}}\gamma_{2}^{d}-2\sqrt{\gamma_{1}^{p}}\gamma_{2}^{p}$$

where γ^d^ and γ^p^ are the dispersion and polar components of the surface tension, respectively. The values of different surface tensions are listed in Table 3. Based on Equations (1) and (2), the calculated value of ω_α_ was found to be ~6.3 indicating that the carbon black particles should be selectively distributed in the continuous PP matrix phase.

The droplet-matrix morphology of this blend was further verified from Kerner’s two phase theoretical model as follows [16]:

(3) $$\frac{E^{\prime }}{{E^{\prime }}_{m}}=\gamma \frac{\left(1-\phi_{i}\right){E^{\prime }}_{m}+\beta \left(\alpha +\phi_{i}\right){E^{\prime }}_{i}}{\left(1+\alpha \phi_{i}\right){E^{\prime }}_{m}+\alpha \beta \left(1-\phi_{i}\right){E^{\prime }}_{i}}$$

where

$$\alpha =\frac{2\left(4-\nu_{m}\right)}{\left(7-5\nu_{m}\right)},\beta =\frac{\left(1+\nu_{m}\right)}{\left(1+\nu_{i}\right)},\gamma =\frac{\left(1+\nu \right)}{\left(1+\nu_{m}\right)}$$

where E′ and ν are the storage modulus and the Poisson ratio of the blend, respectively. The prefix m and i represents the matrix and dispersed phase. The change of Poisson ratio with temperature was obtained from the following equation [16]:

(4) $$\nu \left(T\right)=\gamma \frac{\left\{0.17\log E^{\prime }\left(\mathrm{glass}\right)-\log^{\prime }\left(T\right)\right\}}{\left\{\log E^{\prime }\left(\mathrm{glass}\right)-\log E^{\prime }\left(\mathrm{rubber}\right)\right\}}+0.32$$

**Figure 7:**
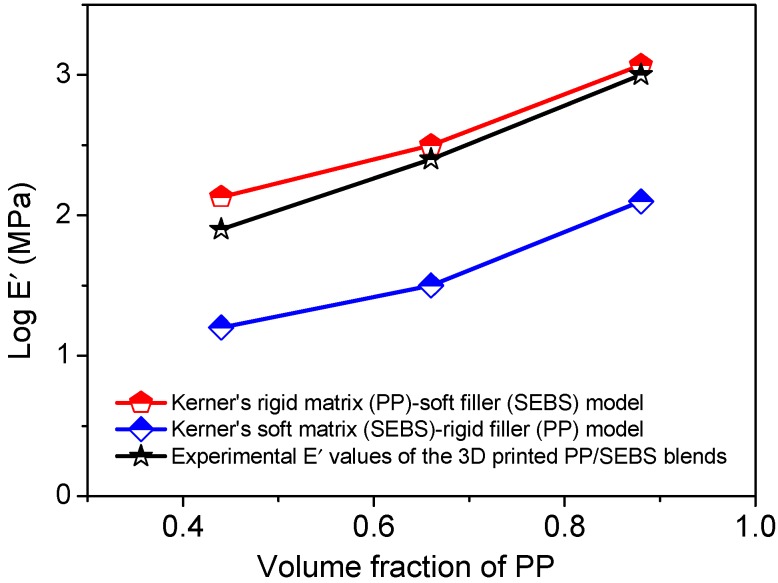
Storage modulus vs. volume fraction of PP. Kerner’s model was applied to predict the droplet-matrix morphology. Experimental storage modulus was compared with the storage modulus obtained using Kerner’s model.

In these blends, PP is the hard phase and SEBS is the soft phase. Figure 7 plots the experimental and theoretical storage modulus for different continuous phases. It can be seen that the experimental storage modulus values of the blends are very close to the predicted Kerner’s hard matrix-soft filler phase, when the PP is considered the continuous matrix phase in this blend.

## 4. Conclusions

In summary, this work demonstrated the successful development of ME3DP compatible thermoplastic elastomeric materials from PP/SEBS compositions based on a facile blending strategy, suitable for commercial FDM 3D printers to produce printed parts containing user-defined regions with good elasticity. It was found that not all of PP/SEBS blends compositions were printable and not all printable compositions could be considered TPEs. 3D printability of TPEs of varying blend compositions was determined by printing standard tensile test specimens. Among the various prepared 3D printable compositions, only the 40 PP/60 SEBS (*w*/*w*) composition exhibited thermoplastic elastomeric behavior. Performance of the 3D printed TPE in terms of mechanical, dynamic mechanical, and rheological properties was compared with the injection molded samples. Tensile strength and Young’s modulus of the 3D printed TPEs were lower than the conventional injection molded samples due to the weak interface between the printed layers. On the other hand, there were no significant changes in the dynamic mechanical and rheological properties of the 3D printed TPE compared with the injection molded samples. At lower frequencies the complex viscosity and storage modulus of the injection molded TPE was higher than the 3D printed TPE. To improve the mechanical performance of the 3D printed TPE, a reinforcing grade of carbon black was used in the range of 0 to 15 phr. Interestingly, at lower loading of carbon black (7.5 phr), adequate improvement of the mechanical, dynamical mechanical, and rheological properties was obtained. However, at 15 phr loading, a reduction of tensile strength and elongation at break were obtained as compared to the 7.5 phr loading. Microstructural analysis revealed that PP formed the continuous matrix with SEBS as the dispersed phase in these blends. This was validated using the theoretical Kerner model. In the case of the filled TPEs, location of carbon black was also theoretically predicted from the wetting parameters to be preferentially located in the continuous PP phase. This work provides a promising pathway for the preparation of new polymeric materials using ME3DP technology.

## Figures and Tables

**Table 1:** Extrusion process parameters of PP/SEBS blends.

	Zone 1	Zone 2	Zone 3	Zone 4	Zone 5	Zone 6	Zone 7	Zone 8
Set (°C)	190	195	200	200	200	200	200	200
Actual (°C)	187	195	197	199	198	197	202	203
Head Pressure (PSI)		50	Extruder Load (%)		27	Extruder Speed (rpm)		190

**Table 2:** List of 3D printing process parameters of PP/SEBS blends.

Layer Thickness (mm)	0.2
Object infill (%)	100
Print temperature (°C)	220
Build-platform temperature (°C)	100
Number of shells	1
Print speed (mm/s)	35
Travel federate (mm/s)	70
G-code nozzle diameter (mm)	0.4
Raster angle (°)	45

**Table 3:** Surface tension of PP and SEBS at room temperature [17,23,24] and the calculated interfacial tension values as per Equation (2).

Component	γ (mN/m)	γ^d^ (mN/m)	γ^p^ (mN/m)
PP	40.3	37.8	2.5
SEBS ^a^	25.54	22.9	2.6
Carbon Black (CB)	99	95.7	3.3
**Composition**	**γ (mN/m) as per Equation (2)**		
PP/SEBS	1.8		
PP/CB	13.2		
SEBS/CB	24.9		

^a^ The polar and dispersive components of surface tension for SEBS was obtained by adopting a similar methodology as shown by [24], where only ethylene-butylene group is considered as it is the major component of block copolymer (SEBS).

## References

[1] Stansbury J.W., Idacavage M.J. (2016). 3D printing with polymers: Challenges among expanding options and opportunities. Dental Mater..

[2] Wang X., Jiang M., Zhou Z., Gou J., Hui D. (2017). 3D printing of polymer matrix composites: A review and prospective. Compos. Part B Eng..

[3] Hart L.R., Li S., Sturgess C., Wildman R., Jones J.R., Hayes W. (2016). 3D Printing of Biocompatible Supramolecular Polymers and their Composites. ACS Appl. Mater. Interfaces.

[4] Muth J.T., Vogt D.M., Truby R.L., Mengüç Y., Kolesky D.B., Wood R.J., Lewis J.A. (2014). 3D Printing: Embedded 3D Printing of Strain Sensors within Highly Stretchable Elastomers. Adv. Mater..

[5] Dizon J.R.C., Espera A.H., Chen Q., Advincula R.C. (2018). Mechanical characterization of 3D-printed polymers. Addit. Manuf..

[6] Ligon S.C., Liska R., Stampfl J., Gurr M., Mülhaupt R. (2017). Polymers for 3D Printing and Customized Additive Manufacturing. Chem. Rev..

[7] Schmid M., Wegener K., Amado A. (2014). Materials perspective of polymers for additive manufacturing with selective laser sintering. J. Mater. Res..

[8] Rocha C.R., Shemelya C.M., Wicker R.B., Perez A.R.T., Roberson D.A., Macdonald E. (2014). Novel ABS-based binary and ternary polymer blends for material extrusion 3D printing. J. Mater. Res..

[9] Fafenrot S., Grimmelsmann N., Wortmann M., Ehrmann A. (2017). Three-Dimensional (3D) Printing of Polymer-Metal Hybrid Materials by Fused Deposition Modeling. Materials.

[10] Banerjee S.S., Bhowmick A.K. (2017). High-temperature thermoplastic elastomers from rubber–plastic blends: A state-of-the-art review. Rubber Chem. Technol..

[11] Banerjee S.S., Bhowmick A.K. (2013). Novel nanostructured polyamide 6/fluoroelastomer thermoplastic elastomeric blends: Influence of interaction and morphology on physical properties. Polymer.

[12] Coran A.Y., Patel R. (1981). Rubber-Thermoplastic Compositions. Part IV. Thermoplastic Vulcanizates from Various Rubber-Plastic Combinations. Rubber Chem. Technol..

[13] Banerjee S.S., Kumar K.D., Sikder A.K., Bhowmick A.K. (2015). Nanomechanics and Origin of Rubber Elasticity of Novel Nanostructured Thermoplastic Elastomeric Blends Using Atomic Force Microscopy. Macromol. Chem. Phys..

[14] Chen Y., Kushner A.M., Williams G.A., Guan Z. (2012). Multiphase design of autonomic self-healing thermoplastic elastomers. Nat. Chem..

[15] Banerjee S.S., Bhowmick A.K. (2015). Dynamic vulcanization of novel nanostructured polyamide 6/fluoroelastomer thermoplastic elastomeric blends with special reference to morphology, physical properties and degree of vulcanization. Polymer.

[16] Banerjee S.S., Bhowmick A.K. (2015). Tailored Nanostructured Thermoplastic Elastomers from Polypropylene and Fluoroelastomer: Morphology and Functional Properties. Ind. Eng. Chem. Res..

[17] Wilkinson A., Clemens M., Harding V. (2004). The effects of SEBS-g-maleic anhydride reaction on the morphology and properties of polypropylene/PA6/SEBS ternary blends. Polymer.

[18] Setz S., Stricker F., Duschek T. (1996). Morphology and mechanical properties of blends of isotactic or syndiotactic polypropylene with SEBS block copolymers. J. Appl. Polym. Sci..

[19] Banerjee S.S., Bhowmick A.K. (2015). Viscoelastic properties and melt rheology of novel polyamide 6/fluoroelastomer nanostructured thermoplastic vulcanizates. J. Mater. Sci..

[20] George S., Ramamurthy K., Anand J., Groeninckx G., Varughese K., Thomas S. (1999). Rheological behaviour of thermoplastic elastomers from polypropylene/acrylonitrile–butadiene rubber blends: Effect of blend ratio, reactive compatibilization and dynamic vulcanization. Polymer.

[21] Banerjee S.S., Kumar K.D., Bhowmick A.K. (2015). Distinct Melt Viscoelastic Properties of Novel Nanostructured and Microstructured Thermoplastic Elastomeric Blends from Polyamide 6 and Fluoroelastomer. Macromol. Mater. Eng..

[22] Banerjee S.S., Janke A., Jehnichen D., Gohs U., Heinrich G. (2018). Influence of electron-induced reactive processing on structure, morphology and nano-mechanical properties of polyamide 6/fluoroelastomer blends. Polymer.

[23] Gong T., Peng S.-P., Bao R.-Y., Yang W., Xie B.-H., Yang M.-B. (2016). Low percolation threshold and balanced electrical and mechanical performances in polypropylene/carbon black composites with a continuous segregated structure. Compos. Part B Eng..

[24] Menke T.J., Funke Z., Maier R.-D., Kressler J. (2000). Surface Tension Measurements on Ethene-Butene Random Copolymers and Different Polypropenes. Macromolecules.

[25] Feng J., Chan C.-M., Li J.-X. (2003). A method to control the dispersion of carbon black in an immiscible polymer blend. Polym. Eng. Sci..

[26] Wu S. (1982). Polymer Interfaces and Adhesion.

[27] O’Donnell H.J., Baird D.G. (1995). In situ reinforcement of polypropylene with liquid-crystalline polymers: Effect of maleic anhydride-grafted polypropylene. Polymer.

